# Derangements of liver tissue bioenergetics in Concanavalin A-induced hepatitis

**DOI:** 10.1186/1471-230X-13-6

**Published:** 2013-01-12

**Authors:** Mariam Al-Shamsi, Allen Shahin, Eric PK Mensah-Brown, Abdul-Kader Souid

**Affiliations:** 1 Department of Immunology, College of Medicine and Health Sciences, United Arab Emirates University, Al Ain, Abu Dhabi, United Arab Emirates (UAE; 2 Department of Anatomy, College of Medicine and Health Sciences, United Arab Emirates University, Al Ain, Abu Dhabi, United Arab Emirates (UAE; 3 Department of Pediatrics, College of Medicine and Health Sciences, United Arab Emirates University, Al Ain, Abu Dhabi, United Arab Emirates (UAE

**Keywords:** Concanavalin A (ConA), Hepatitis, Caspase, AST, ALT, IFN-γ

## Abstract

**Background:**

A novel *in vitro* system was employed to investigate liver tissue respiration (mitochondrial O_2_ consumption) in mice treated with concanavalin A (Con A). This study aimed to investigate hepatocyte bioenergetics in this well-studied hepatitis model.

**Methods:**

C57Bl/6 and C57Bl/6 *IFN-γ*^*−/−*^ mice were injected intravenously with 12 mg ConA/kg. Liver specimens were collected at various timepoints after injection and analyzed for cellular respiration and caspase activation. Serum was analyzed for interferon-gamma (IFN-γ) and aminotransferases. Fluorescence activated cell sorting analysis was used to determine the phenotype of infiltrating cells, and light and electron microscopy were used to monitor morphological changes. Phosphorescence analyzer that measured dissolved O_2_ as function of time was used to evaluate respiration.

**Results:**

In sealed vials, O_2_ concentrations in solutions containing liver specimen and glucose declined linearly with time, confirming zero-order kinetics of hepatocyte respiration. O_2_ consumption was inhibited by cyanide, confirming the oxidation occurred in the respiratory chain. Enhanced liver respiration (by ≈68%, p<0.02) was noted 3 hr after ConA treatment, and occurred in conjunction with limited cellular infiltrations around the blood vessels. Diminished respiration (by ≈30%, *p*=0.005) was noted 12 hr after ConA treatment, and occurred in conjunction with deranged mitochondria, areas of necrosis, and prominent infiltrations with immune cells, most significantly, CD3^+^NKT^+^ cells. Increases in intracellular caspase activity and serum IFN-γ and aminotransferase levels were noted 3 hr after ConA treatment and progressed with time. The above-noted changes were less pronounced in C57Bl/6 IFN-γ^−/−^ mice treated with ConA.

**Conclusions:**

Based on these results, liver tissue bioenergetics is increased 3 hr after ConA exposure. This effect is driven by the pathogenesis of the disease, in which IFN-γ and other cytokines contribute to. Subsequent declines in liver bioenergetics appear to be a result of necrosis and active caspases targeting the mitochondria within hepatocytes.

## Background

The term “cellular bioenergetics” describes the biochemical processes involved in energy metabolism (energy conversion or transformation), and the term “cellular respiration” (mitochondrial oxygen consumption) describes the delivery of metabolites and oxygen (O_2_) to the mitochondria, the oxidation of reduced metabolic fuels with the passage of electrons to O_2_, and the synthesis of ATP. Impaired cellular bioenergetics or respiration thus entails an interference with any of these metabolic processes.

Concanavalin A (ConA) is a plant lectin from seeds of *Canavalia ensiformis* [Jack bean] that serves as a polyclonal T-cell mitogen and is also used to induce hepatitis in mice. Murine ConA-induced hepatitis is a well-studied model that mimics human liver viral infections [1,2]. The liver injury is typically noted within 3 hr of intravenous injection of ConA (12 mg/kg) and progresses with time [2]. Activation and recruitment of natural killer T (NK T) and other cells of the innate immune system are early events that, in turn, lead to secretion of inflammatory cytokines, such as interferon-γ (IFN-γ), tumor necrosis factor (TNF)-α, interleukin (IL)-12, and IL-18 [3]. This biochemical burst of inflammatory mediators targets multiple organs, including the liver. Its outcome is a hepatotoxicity that is characterized by mononuclear cellular infiltration and foci of necrosis [4].

The potential role of NK T cells in the pathogenesis of ConA-induced hepatitis is confirmed by the observation that mice lacking NK T cells and those treated with anti-IFN-γ antibody (to counter this essential inflammatory mediator released by NK T cells) are both resistant to ConA-induced hepatitis. In addition, targeting NK T cells with glucocerebroside, a naturally occurring glycolipid that inhibits NK T cell proliferation, ameliorates ConA-induced hepatitis [5]. These findings suggest the pathological outcome of the liver in ConA-treated animals relies on initial activation of innate immune components, such as NK T cells, followed by subsequent activation and recruitment of effector immune cells, including macrophages and T-cells. We confirm here that the pathology of concanavalin A-induced hepatitis is dependent on activated immune cells especially NK T cells and the secretion of cytokines, including IFN-γ, and mice in which the cytokine is genetically deleted develop a milder form of the disease.

ConA-induced hepatitis is associated with caspase activation [6]. Caspase-3, a cysteine aspartate-directed protease and a family member of the IL-1β-converting enzyme (ICE), is the key executer of apoptosis. This enzyme is involved in proteolysis of several proteins, including poly (ADP ribose) polymerase; it cleaves at the C-terminal to Asp^216^ in the DEVD (asp-glu-val-asp) sequence. This 4-amino-acid motif has been utilized for the highly-specific caspase-3 substrate, Ac-DEVD-AMC (*N*-acetyl-DEVD-7-amino-4-methyl-coumarin). Caspase-3 cleaves the tetrapeptide between D and AMC, releasing the fluorogenic moiety 7-amino-4-methylcoumarin (AMC) that can be separated on HPLC and detected by fluorescence [7].

The status of liver tissue mitochondrial O_2_ consumption in ConA-induced hepatitis is unknown. In this study, compound alterations in liver tissue respiration were noted early in the course of the induced hepatitis. The inflammatory cytokines, including IFN-γ appear to contribute to these deleterious changes in hepatocyte energy metabolism.

The effect of inflammatory bursts on hepatocyte bioenergetics is unknown, especially in ConA-induced hepatitis. In theory, inflammatory cytokines are expected to enhance cellular energy metabolism; however, this phenomenon has not yet been investigated and answers are needed. This present study attempts to address some of these queries. Measuring liver tissue respiration, using the principle that O_2_ quenches the phosphorescence of palladium (Pd) II-*meso*-tetra-(4-sulfonatophenyl)-tetrabenzoporphyrin, has been recently reported [8-10]. This analytical tool is used here to monitor liver tissue O_2_ consumption in ConA-induced hepatitis in mice.

## Methods

### Reagents

The Pd(II) complex of *meso*-tetra-(4-sulfonatophenyl)-tetrabenzoporphyrin (Pd phosphor) was obtained from Porphyrin Products (Logan, UT). Dactinomycin (actinomycin D, MW ≈ 1255) was purchased from Merck (Whitehouse Station, NJ). A lyophilized powder of caspase inhibitor I (zVAD-fmk, MW ≈467.5) was purchased from Calbiochem (La Jolla, CA). Ac-DEVD-AMC (MW ≈729.6) and caspase-3 (molecular mass ≈30.5 kDa, heterodimer active human recombinant) were purchased from Axxora LLC (San Diego, CA). Glucose [anhydrous], ConA (from *Canavalia ensifomis,* Jack bean, Type IV-S), bovine serum albumin (free of endotoxin and fatty acids), and remaining reagents were bought from Sigma-Aldrich (St. Louis, MO).

Dactinomycin solution was made fresh in dH_2_O; its concentration was determined by absorbance at 440 nm, using an extinction coefficient of 24,450 M^-1^ · cm^-1^[7]. The zVAD-fmk solution (2.14 mM) was made by dissolving 1.0 mg of zVAD-fmk in 1.0 ml of dimethyl sulfoxide and stored at −20°C. The Ac-DEVD-AMC caspase substrate was dissolved in dimethyl sulfoxide at a concentration of 6.85 mM and stored at −20°C in small aliquots. Phosphate-buffered saline (PBS) with glucose (137 mM NaCl, 2.7 mM KCl, 4.3 mM Na_2_HPO_4_, 1.4 mM KH_2_PO_4_, and 5 mM glucose; pH 7.4) was made fresh. ConA (5 mg) was suspended in 3 ml sterile PBS (f/c = ~1.67 mg/ml); aliquots were stored at −20°C. Pd phosphor solution (2.5 mg/ml = 2 mM) was prepared in dH_2_O and stored in aliquots at −20°C. NaCN solution (1.0 M) was prepared in dH_2_O; the pH was adjusted to ~7.0 with 12 N HCl and stored at −20°C.

### Mice

Male C57Bl/6 and C57Bl/6 IFN-γ^−/−^ mice (8-12-wk-old; weight ≈18-22 g) used in this study were maintained at an animal facility that was in compliance with NIH guidelines (http://grants.nih.gov/grants/olaw/references/phspol.htm). The mice were purchased from The Jackson Laboratory (Bar Harbor, ME). All mice were housed in rooms maintained at 22°C with ~60% relative humidity and a 12-hr light/dark cycle. All mice had *ad libitum* access to standard rodent chow and filtered water. All protocols here received approval from the Animal Ethics Committee-United Arab Emirates University - College of Medicine and Health Sciences.

For these studies, experimental mice were given a single intravenous injection (12 mg/kg body weight in a total volume of 300 μl) of ConA in PBS or PBS alone in the tail vein. At 0–12 hr after the treatment, mice were euthanized by intra-peritoneal injection of 100 μl/10 g (BW) of urethane (using 20% solution [w/v] in 0.9% NaCl) and blood was collected from the sino-orbital orifice; serum was subsequently prepared by standard protocols. At necropsy, liver specimens were collected for analyses.

### Liver tissue

Liver specimens (18–30 mg/mouse) were excised using a sharp pair of scissors (Moria Vannas Wolg Spring, cat. # ST15024-10, Cambridge, UK). Following protocols previously outlined [8,9], the specimens were *immediately* immersed in ice-cold oxygenated Krebs-Henseleit buffer (115 mM NaCl, 25 mM NaHCO_3_, 1.23 mM NaH_2_PO_4_, 1.2 mM Na_2_SO_4_, 5.9 mM KCl, 1.25 mM CaCl_2_, 1.18 mM MgCl_2_, and 6 mM glucose [pH 7.2]), weighed and then placed in 1.0 ml Krebs buffer containing 0.5% fat-free bovine albumin and 3 μM Pd phosphor for O_2_ measurements. Unless otherwise noted, the time period between specimen collection and start of O_2_ measurement was < 5 min. Where stated, specimens were incubated *in vitro* at 37°C in Krebs-Henseleit solution gassed with 95% O_2_:5% CO_2_ prior to O_2_ measurements.

Specimens were also fixed in 4% phosphate-buffered paraformaldehyde and embedded in paraffin wax blocks. Sections of the fixed liver pieces (of 5–7 μm thickness) were stained with haematoxylin and examined under a light microscope. Sections of the harvested liver were also placed in Karnovsky’s solution and processed for electron microscopy [8].

### Oxygen measurements

A phosphorescence oxygen analyzer was used to monitor O_2_ consumption by liver specimens [8,9]. O_2_ detection was performed with the aid of Pd phosphor that had an absorption maximum at 625 nm and a phosphorescence maximum at 800 nm. Samples were exposed to light flashes (600 per min) from a pulsed light-emitting diode array with peak output at 625 nm (OTL630A-5-10-66-E, Opto Technology, Inc., Wheeling, IL). Emitted phosphorescent light was detected by a Hamamatsu photomultiplier tube (928) after first passing it through a wide-band interference filter centered at 800 nm. The amplified phosphorescence decay was digitized at 1.0 MHz by a 20-MHz A/D converter (Computer Boards, Inc., Mansfield, MA).

A program was developed using Microsoft Visual Basic 6, Microsoft Access Database 2007, and Universal Library components (Universal Library for Measurements Computing Devices; http://www.mccdaq.com/daq-software/universal-library.aspx). It allowed direct reading from the PCI-DAS 4020/12 I/O Board (PCI-DAS 4020/12 I/O Board; http://www.mccdaq.com/ pci-data-acquisition/PCI-DAS4020-12.aspx). The pulse detection was accomplished by searching for 10 phosphorescence intensities >1.0 volt (by default). Peak detection was accomplished by searching for the highest 10 datapoints of a pulse and choosing the datapoint closest to the pulse decay curve [10].

The phosphorescence decay rate (1/τ) was characterized by a single exponential; I = Ae^-*t*/τ^, where I = Pd phosphor phosphorescence intensity. The values of 1/τ were linear with dissolved O_2_: 1/τ = 1/τ^o^ + *k*_*q*_[O_2_, where 1/τ = the phosphorescence decay rate in the presence of O_2_, 1/τ^o^ = the phosphorescence decay rate in the absence of O_2_, and *k*_q_ = the second-order O_2_ quenching rate constant in sec^-1^ · μM^-1^[11].

Liver tissue respiration was measured at 37°C in 1 ml sealed vials. Mixing was carried out with the aid of parylene-coated stirring bars. In vials sealed from air, [O_2_] decreased linearly with time, indicating the kinetics of mitochondrial O_2_ consumption was zero-order. The rate of respiration (*k*, in μM O_2_/min) was thus the negative of the slope d[O_2_]/d*t*. Sodium cyanide (NaCN) inhibited respiration, confirming that O_2_ was being consumed in the mitochondrial respiratory chain (Additional file 1: Figure S1).

### Calibration with β-glucose plus glucose oxidase

The calibration reaction contained PBS with 3 mM Pd phosphor, 0.5% fat-free albumin, 50 mg/ml glucose oxidase and various concentrations of β-glucose. The values of 1/t were linear with [β-glucose]; the value of *k*_q_ was the negative of the slope (*k*_q_ = 101.1 sec^-1^ · μM^-1^). The value of 1/τ for air-saturated solution (without glucose) was 28,330 sec^-1^ (coefficient of variation, C_v_ = 12%) and for O_2_-depleted solution (with 500 mM β-glucose, 1/τ_o_) 2,875 sec^-1^ (C_v_ = 1%). The high values of C_v_ for the air-saturated solutions were due to the lower

phosphorescence intensities with high [O_2_] (little light reaching the photomultiplier tube). O_2_ concentration was calculated using, 1/τ = 1/τ^o^ + *k*_*q*_[O_2_].

Dissolved O_2_ is expressed in mm Hg, ml O_2_/L, mg O_2_/L, or mmol/L (mM). For conver-sion: A partial pressure of oxygen (*P*O_2_) of 1.0 mm Hg = 0.03 ml O_2_/L; 1.0 ml O_2_/L = 1.4276 mg O_2_/L; 1.0 mg O_2_/L = 1000/32 μM. In freshwater at 760 mm Hg and 20°C, dissolved [O_2_] is 9.1 mg/L, or 284 μM. Using a Clark electrode, the *P*O_2_ of our reaction mixture (PBS with 10 mM glucose, 3.0 μM Pd phosphor and 0.5% fat-free bovine serum albumin) was 170.5 [± 6.6] mm Hg (n = 4), or 228 [± 9] μM. The 56 mm Hg difference between [O_2_] in freshwater and the Pd solution reflects the effect of salinity on dissolved O_2_.

### Serum IFN-γ and aminotransferases

Serum IFN-γ levels were determined using a sandwich ELISA DuoSet ELISA Develop-ment (mouse IFN-γ) kit (R&D Systems, Minneapolis, MN), according to manufacturer protocols. IFN-γ concentrations (in pg/ml) were extrapolated from a standard curve generated in parallel using kit-provided recombinant IFN-γ. The level of sensitivity of the kit was 20 pg/ml.

Serum alanine aminotransferase (ALT) activity was determined on a Beckman Coulter Synchron® System (Brea, CA); 150 μl serum/mouse were used in a reaction that contained L-alanine, α-ketoglutarate, NADH and lactate dehydrogenase. NADH oxidation, calculated from the change in absorbance at 340 nm, is proportional to the transaminase activity and the basis for the calculation of ALT activity/sample. The level of sensitivity of the assay was 5 IU/L. Serum aspartate aminotransferase (AST) activity was determined as above, in a reaction that contained L-aspartate, α-ketoglutarate, NADH, and malate dehydrogenase. The level of sensitivity of the assay was 5 IU/L.

### Intracellular caspase activity

Liver specimens (40–55 mg) were incubated at 37°C in oxygenated KH buffer containing 74 μM Ac-DEVD-AMC with and without 43 μM zVAD-fmk (final volume = 0.5 ml) for 30 min. At the end of the incubation period, the suspension was sonicated on ice for 60 sec and passed 10 times through a 27-gauge needle. The cleavage reaction was quenched with tissue disruption, as caspases became inactive due to the dilution. The supernatants were then collected by centrifugation (~12300 × *g* for 15 min, 25°C), separated via HPLC and analyzed for free AMC as described below [7].

### HPLC

The analysis was performed on a Waters 1525 reversed-phase HPLC system (SpectraLab Scientific Inc, Alexandria, VA) that consisted of a manual injector, pump, and fluorescence detector. The excitation wavelength used was 380 nm and the emission wavelength 460 nm. Solvent A was HPLC-grade CH_3_CN:H_2_O [1:3, v/v], and Solvent B was dH_2_O (isocratic). The Ultrasphere IP column (4.6 × 250 mm, Beckman, Brea) was operated at 25°C at 1.0 ml/min (0.5 ml/min of each pump). The run time was 30 min.

### Phenotyping of infiltrating mononuclear cells by fluorescence activated cell sorting analysis (FACS) analysis

Liver fragments were placed in RPMI with 5% fetal calf serum (FCS; Hyclone, Logan, UT) and 25 mM HEPES, and passed through 200-gauge stainless steel mesh. The pellets were then collected by centrifugation (~500 × *g* at 4°C for 10 min), suspended in 40% Percol®, and layered on the top of 70% Percol®. The solutions were then centrifuged at ~100 × *g* and 25°C for 20 min. The mononuclear cells were collected from the interface, washed twice with RPMI, and re-suspended in RPMI at 3 × 10^5^/ml.

For immunophenotyping, the fluorescent-labeled antibodies fluorescein isothiocyanate (FITC)-conjugated rat anti-mouse CD3, CD4, and CD45 and phycoerythrin (PE)-conjugated rat anti-mouse CD8, NK T, and CD11b were used (all were purchased from eBioscience, San Diego, CA). To avoid non-specific binding, 1 × 10^6^ cells/mouse were incubated with 20% FCS for 10 min at 4°C. The cells were then gently washed with PBS supplemented with 5% FCS and the resultant pellet treated with 100 μl aliquots (from 3 μg/ml stocks) each of a given pair of antibodies, i.e., FITC-anti-CD4 + PE-anti-CD8, FITC-anti-CD3 + PE-anti-NK/NKT, or FITC-anti-CD45 + PE-anti-CD11b. The cells were then incubated for 35 min at 4°C in the dark. Thereafter, the cells were washed twice with PBS-FCS and then re-suspended at 10^6^ cells/tube in 0.5 ml PBS-FCS. Samples were then analyzed via flow cytometry using a FACSort system (Becton Dickinson, Franklin Lakes, NJ) and associated CellQuest software. A minimum of 10,000 events per sample were acquired.

### Statistical analysis

The Student’s *t*-test of difference of means was used to compare treated and untreated samples. The threshold of significance was taken to be p < 0.05.

## Results

### Hepatocyte oxygen consumption in C57Bl/6 mice treated with ConA

**Figure 1:**
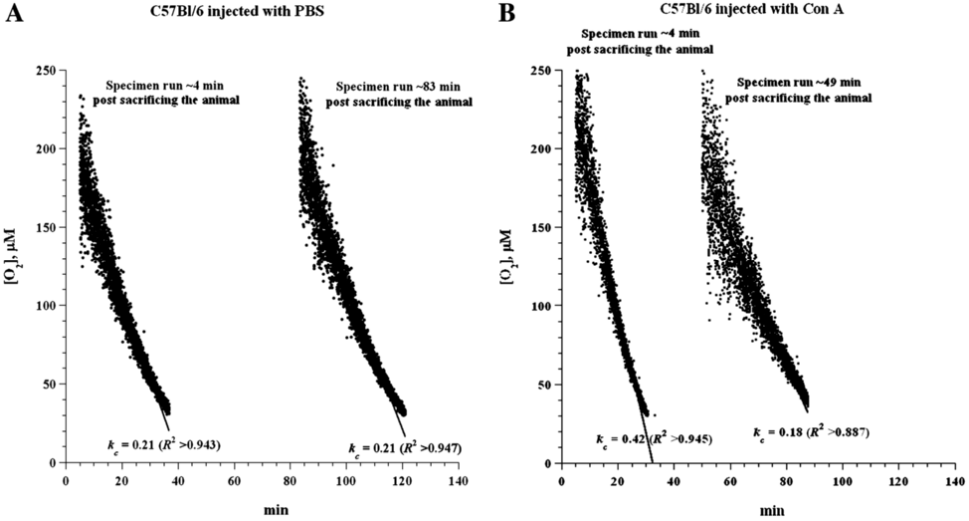
**Liver respiration 3 hr after ConA injection (immediately post-tissue collection and after *in vitro *incubation).** C57Bl/6 wild-type mice were injected either with PBS (**Panel A**) or with 12 mg ConA/kg (**Panel B**). Two liver specimens were then collected per mouse at 3 hr post-injection; minute zero corresponds to the time of sacrifice of an animal. For untreated mice, one specimen (25 mg) was run at *t* = 4 (immediately post-collection) and one (27 mg) at *t* = 83 min. For the treated mice, one specimen (20 mg) was run at *t* = 4 (immediately post-collection) and one (24 mg) at *t* = 49 min. The lines shown are linear fits. Rates of respiration (*k*_*c*_, in μM O_2_ min^-1^ · mg^-1^) and *R*^2^ values are shown at the bottom of the runs.

Figure 1 depicts a representative experiment of hepatocyte respiration in ConA-treated and untreated C57Bl/6 mice. One mouse was injected with PBS (Panel A) and one with 12 mg ConA/kg per body weight (Panel B). Two liver specimens were collected from each mouse 3 hr after injection. For the untreated mouse, one specimen was run immediately and one after ~83 min of *in vitro* incubation at 37°C in Krebs-Henseleit solution gassed with 95% O_2_:5% CO_2_. The rate of respiration (*k*_*c*_, μM O_2_ min^-1^ · mg^-1^) in both specimens was 0.21, confirming stability of the liver specimens *in vitro*[8,9]. For the treated mouse, one specimen was also run immediately post collection and one after ~49 min of *in vitro* incubation as above. The values of *k*_*c*_ were 0.42 (2-fold increment) and 0.18 (similar to the control) respectively. These results demonstrate accelerated rate of liver tissue O_2_ consumption 3 hr after ConA injection*.* In another experiment, the values of *k*_*c*_ for the untreated mouse were 0.20 at *t* = 5 min (immediately post specimen collection) and 0.24 at *t* = 83 min (after 83 min incubation *in vitro* as above); the corresponding values for the treated mouse were 0.34 at *t* = 3 min (a 70% increment) and 0.14 at *t* = 59 min. In a third experiment, the values of *k*_*c*_ for the untreated mouse were 0.25 at *t* = 5 min and 0.23 at *t* = 58 min; corresponding values for the treated mouse were 0.34 at *t* = 5 min (36% increment) and 0.20 at *t* = 75 min. Thus, for untreated mice, values of *k*_*c*_ (mean ± SD) were 0.22 ± 0.02 (n = 6). For treated mice, values of *k*_*c*_ for specimens run immediately were 0.37 ± 0.05 (n = 3, a 68% increase, p = 0.015) and for specimens incubated *in vitro* before the run, 0.17 ± 0.03 (n = 3, a 54% less, p = 0.005).

In a total of eight independent experiments, the values of *k*_*c*_ in untreated mice were 0.234 ± 0.024 (n = 8) and in treated mice 3 hr after ConA injection (12 mg/kg) 0.300 ± 0.066 (n = 8, p < 0.05). The variability in the values of *k*_*c*_ in treated mice (coefficient of variation = ~22% in ConA- injected mice compared to ~10% in untreated mice) likely reflected the heterogeneity of the liver involvement and the variation of its progress with time.

**Figure 2:**
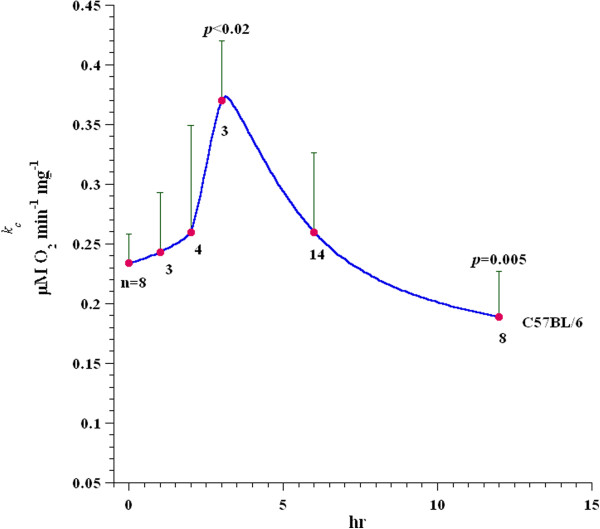
**Time-dependent changes in liver respiration in ConA hepatitis in C57Bl/6 mice.** C57Bl/6 mice (wild-type) were injected with PBS or 12 mg ConA/kg. Liver tissue specimens were then collected at the indicated timepoints and processed for O_2_ measurements. Values shown are the mean (± SD), with the values of “n” underneath each timepoint. The p-values at 3 and 12 hr (compared to 0 hr) are also shown; remaining values were insignificant.

Changes in liver tissue respiration during the course of ConA-induced hepatitis are summarized in Figure 2. As discussed above, the rate of respiration (*k*_c_) significantly increased at 3 hr (p < 0.02) and decreased at 12 hr (p = 0.005, see also Table 1). By contrast, the values of *k*_c_ at 1, 2, and 6 hr after ConA injection did not significantly differ from untreated mice (also see Table 2). Liver tissue respiration was not altered by *in vitro* addition of ConA (Additional file 1: Figure S2).

**Table 1:** Liver tissue respiration in samples harvested from ConA-treated mice after 12 hr

**Strain**	**Treatments**	***k***_**c**_	
		**(μM O**_**2**_**min**^**-1**^**· mg**^**-1**^**)**	
		mean ± SD (n)	range
C57Bl\6 (wild-type)	PBS (300 μl)	0.26 ± 0.04 (5)	0.22 - 0.32
	ConA (12 mg/kg)	^*^0.18 ± 0.03 (5)	0.13 - 0.20

“n” represents number of independent experiments. *Value significantly different from control at p ≤ 0.005.

**Table 2:** **Liver tissue respiration in tissues collected from ConA-treated C57Bl/6 wild-type and *IFN-γ***^***−/−***^**mice 7 hr after treatment**

**Strain**	**Treatment**	***k***_**c**_
		**(μM O**_**2**_**min**^**-1**^**· mg**^**-1**^**)**
C57Bl\6	PBS (180 μl)	0.27 ± 0.13 (11)
C57Bl\6	ConA (12 mg/kg)	0.26 ± 0.07 (3)
C57Bl\6 *IFN-γ*^−/−^	ConA (12 mg/kg)	0.25 ± 0.10 (3)

“n” represents number of independent experiments. Values shown are mean ± SD.

### Hepatocyte oxygen consumption in C57Bl/6 *IFN-γ*^*−/−*^ mice treated with ConA/kg

**Figure 3:**
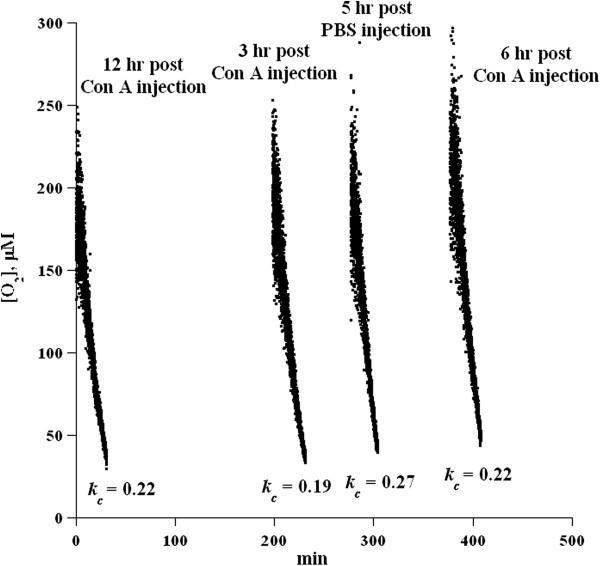
**Time-dependent changes in liver respiration in ConA hepatitis in C57Bl/6 *IFN-γ***^***−/−***^**mice.** Mice were injected with 20 mg ConA/kg and liver specimens (24–29 mg) were collected for O_2_ measurements at 3, 6, and 12 hr post-injection. Control mice were injected with PBS. Rates of respiration (*k*_*c*_, in μM O_2_ min^-1^ · mg^-1^) are shown at the bottom of the runs. All four runs were performed on one instrument. One animal was sacrificed at-a-time in the following sequence: 12 hr post-injection, 3 hr post-injection, PBS, and then 6 hr post-injection. Minute zero corresponds to sacrifice of the first animal.

C57Bl/6 *IFN-γ*^*−/−*^ mice were injected with 20 mg ConA/kg or PBS and then liver pieces were collected 3, 6, and 12 hr post-injection. The mean *k*_c_ value (in μM O_2_ min^-1^ · mg^-1^) for uninjected mouse was 0.27. The corresponding *k*_c_ values for injected mice at 3, 6, and 12 hr post-injection were 0.19, 0.22, and 0.22, respectively (Figure 3 and Table 2). Of note, additions of exogenous IFN-γ (10, 50 or 100 ng/mL) resulted in inhibition of liver tissue respiration (Additional file 1: Figure S3).

### Serum IFN-γ in C57Bl/6 mice treated with ConA

**Figure 4:**
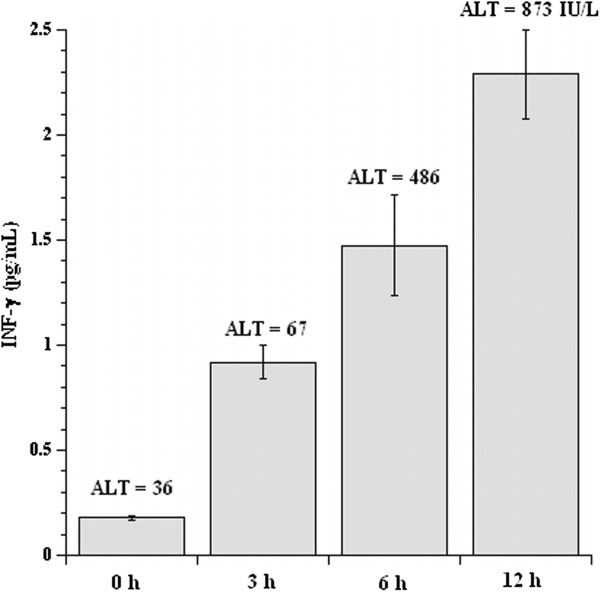
**Serum IFN-γ and ALT in untreated and ConA-treated C57Bl/6 mice.** C57BL/6 mice were injected with PBS or 12 mg ConA/kg. Serum was collected 3, 6, and 12 hr post- injection and processed for IFN-γ. IFN-γ values shown are mean (± SD) of three determinations for each sample (n = 3 animals/sample). ALT activities at 0, 3, 6, and 12 hr are from a single experiment (see Results).

Serum IFN-γ levels were determined in untreated and ConA-treated (12 mg/kg) C57Bl/6 mice at 0, 3, 6, and 12 hr post-injection. The results of three independent experiments are shown in Figure 4. Relative to the values seen in untreated animals, elevated levels of IFN-γ were noted at 3 hr after ConA injection, and these increased with time.

### Serum ALT and AST in C57Bl/6 and C57Bl/6 *IFN-γ*^*−/−*^ mice treated with ConA

Serum ALT activity was determined in untreated and ConA-treated (12 mg/kg) C57Bl/6 mice at 0, 3, 6, and 12 hr post-injection. Elevated levels of ALT were noted at 3 hr after ConA and these increased with time (Figure 4). In three independent experiments, serum ALT levels 12 hr after ConA were 440 ± 393 IU/L (mean ± SD, n = 3) and AST 768 ± 528 (n = 3).

For C57Bl/6 IFN-γ^−/−^ mice, ATL activities were 45, 96, and 32 IU/L at the 0, 3 and 6 hr time points, respectively. The corresponding values for AST were 157, 259, and 228 IU/L. At 12 hr, the values of ALT were 172 ± 154 IU/L (n = 3) and AST 394 ± 198 IU/L (n = 3).

Serum ALT activities in PBS-treated C57Bl/6 mice at 0 and 12 hr were, respectively, 30 and 30 U/L; serum IFN-γ levels in PBS-treated C57Bl/6 mice at 0 and 12 hr were non-measurable. Serum ALT activities in PBS-treated C57Bl/6 IFN-γ^−/−^ mice at 0 and 12 hr were, respectively, 35 and 46 U/L.

### Caspase activation in C57Bl/6 mice treated with ConA

Caspase activity in liver tissue was monitored in untreated and ConA-treated (12 mg/kg) mice at 3, 6, and 12 hr post-injection, using the caspase-3 substrate analogue Ac-DEVD-AMC. Liver specimens were incubated with 74 μM Ac-DEVD-AMC in the presence and absence of the pan-caspase inhibitor zVAD-fmk. Caspases cleaved Ac-DEVD-AMC, releasing the fluorogenic moiety AMC; post-sample disruption, AMC was separated on HPLC (*R*_t_ ~18 min) and detected by fluorescence.

**Figure 5:**
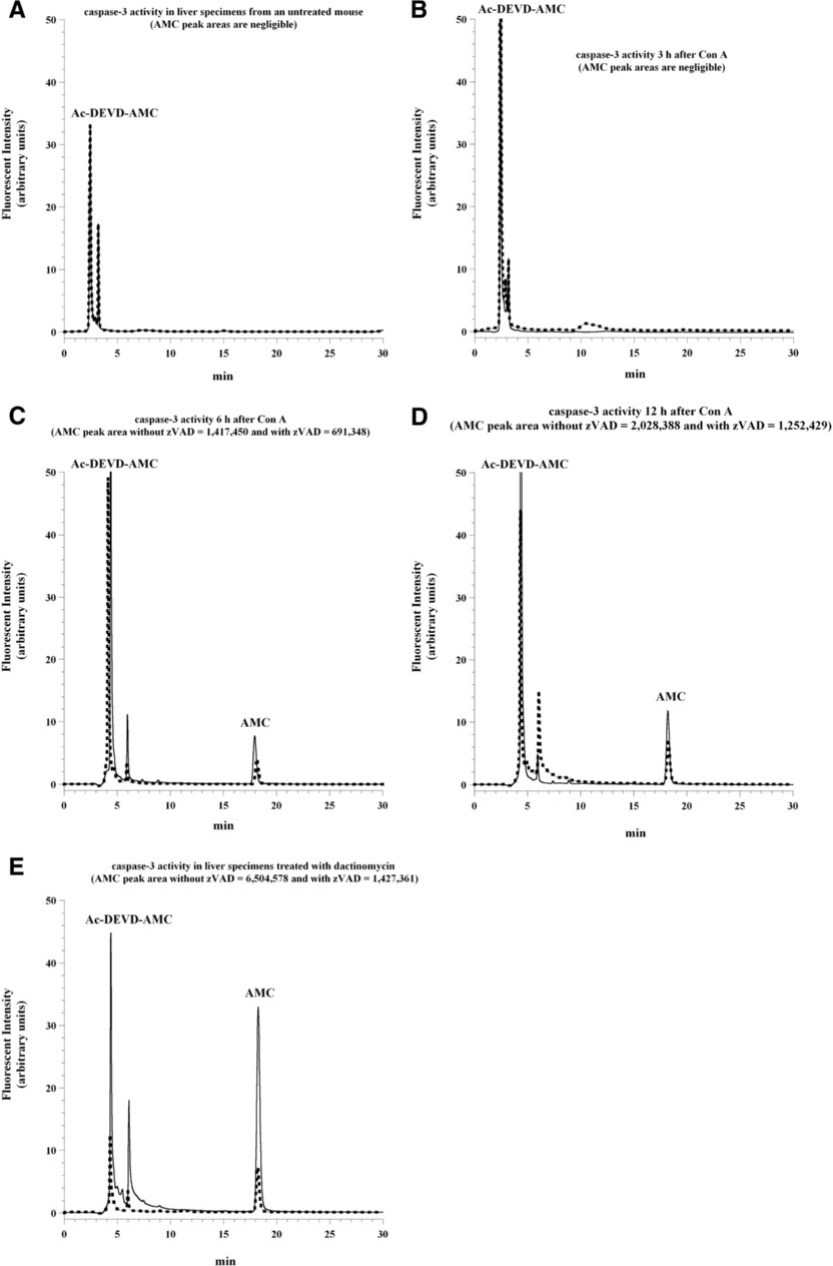
**Liver tissue caspase activation in untreated and ConA-treated C57Bl/6 mice.** C57BL/6 mice were injected with **(A)** PBS or **(B-D)** 12 mg ConA/kg. Liver specimens were collected **(B)** 3, **(C)** 6, or **(D)** 12 hr post-injection and processed immediately for caspase activity. For each condition, liver specimens were incubated at 37°C in oxygenated KH buffer in the presence of 74 μM Ac-DEVD-AMC with (dashed line) and without (solid line) 43 μM zVAD-fmk for 30 min. The cleavage reaction was quenched with tissue disruption. The supernatants were separated via HPLC and analyzed for Ac-DEVD-AMC (*R*_t_, ~3 min) and free AMC (*R*_t_, ~18 min) peaks as described in the Materials and Methods. In **Panel E**, liver specimens were treated *in vitro* with 8 μM dactinomycin for 60 min prior to the addition of Ac-DEVD-AMC with (dashed line) and without (solid line) zVAD-fmk.

AMC peak was neither detected in the untreated mouse (Figure 5A) nor in the ConA-treated mouse at 3 hr (Figure 5B). By contrast, an AMC peak was detected at 6 and 12 hr after ConA injection. At 6 hr (Figure 5C), the AMC peak area (arbitrary units) without zVAD-fmk was 1,417,450 and with zVAD-fmk was 691,348 (~51% inhibition). At 12 hr (Figure 5D), the AMC peak area without zVAD-fmk was 2,028,388 and with zVAD-fmk 1,252,429 (~38% inhibition). These results demonstrate rapid uptake and hydrolysis of Ac-DEVD-AMC by active caspases in hepatocytes. Residual caspase activity in the presence of zVAD-fmk was due to the simultaneous addition of Ac-DEVD-AMC (added in excess) and zVAD-fmk. The free AMC moieties were negligible in experiments where Ac-DEVD-AMC was added 10 min after addition of zVAD-fmk (data not shown). In another two independent experiments, the AMC peak was detected at 3 hr and increased linearly at 12 hr (*R*^*2*^ >0.999 and >0.995).

For comparison, liver tissue specimens were also treated *in vitro* with 8 μM dactinomycin, a cytotoxic agent that intercalates between DNA base pairs and activates caspases [7]. The AMC peak area in the presence of dactinomycin alone was 6,504,578 and in the presence of dactinomycin + zVAD-fmk was 1,427,361 (78% inhibition, Figure 5E).

### Light and electron microscopy of tissues from C57Bl/6 and C57Bl/6 IFN-γ^−/−^ mice

**Figure 6:**
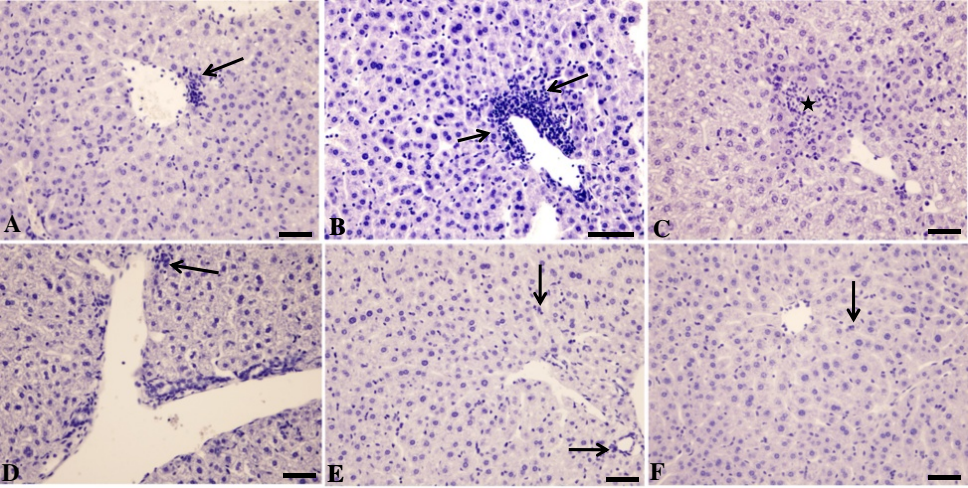
**Micrographs of hematoxylin-stained liver sections from untreated and ConA-treated C57Bl/6 and C57Bl/6 *IFN-γ***^***−/−***^**mice.** Samples are representative of those from **(A, B, C)** wild-type and from **(D, E, F)***IFN-γ*^*−/−*^ C57Bl/6 mice, each of which had been injected with 12 mg ConA/kg. Livers were then collected at **(A, D)** 3, **(B, E)** 6, and **(C, F)** 12 hr post-injection. Note the accumulation of mononuclear cells in the wild-type mice at 3 and (more profusely) 6 hr (arrow), and the solitary cells in the *IFN-γ*^*−/−*^ mice (even 6 hr after injection). Infiltration is found around vessels and sinusoids in both strains. At 12 hr post-injection, areas of necrosis (star) filled with mononuclear cells become apparent in the liver of wild-type mice, but livers of *IFN-γ*^*−/−*^ mice remained unaffected. Bar = 50 μm.

Figure 6 shows the liver histology in ConA-injected (12 mg/kg) C57Bl/6 and C57Bl/6 IFN-γ^−/−^ mice at 3, 6 and 12 hr. In C57Bl/6 mice, mononuclear cell accumulation around blood vessels was noted 3 and 6 hr after the ConA administration. More prominent mononuclear cell infiltration around blood vessels and into the tissues and areas of necrosis were noted 12 hr after the injection in C57Bl/6, but not in C57Bl/6 IFN-γ^−/−^, mice. Overall, the liver architecture was relatively preserved in the C57BL/6 IFN-γ^−/−^ mice.

**Figure 7:**
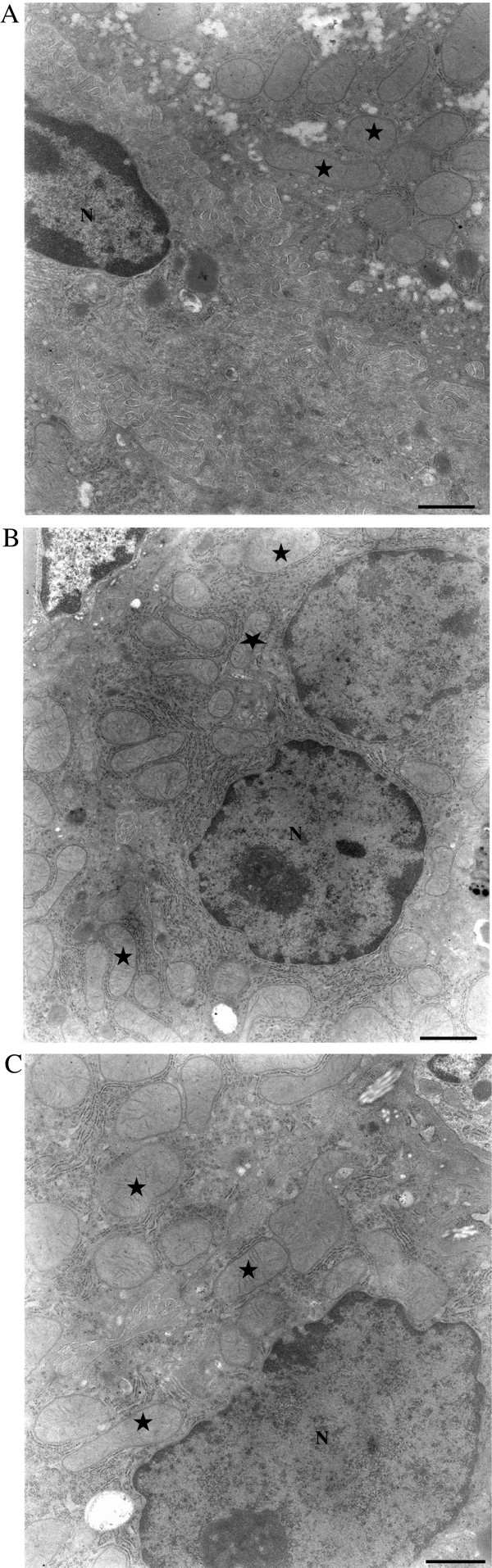
**Electron micrographs of hepatocytes from untreated and ConA-treated C57Bl/6 mice.** Hepatocytes were obtained from **(A)** normal untreated and ConA-injected C57Bl/6 mice **(B)** 3 and **(C)** 6 hr post-injection. A representative micrograph is shown for each timepoint. Note the nuclei and mitochondria in each of the micrographs are normal. Bar = 0.3 μm.

Electron micrographs of the hepatocytes showed a normal presence of nuclei and mitochondria in untreated and ConA-treated (12 mg/kg) C57Bl/6 mice at 3 and 6 hr after injection (Figure 7).

The histopathology of liver sections obtained from uninjected or PBS-injected C57Bl/6 and from uninjected or PBS-injected C57Bl/6 IFN-γ^−/−^ mice showed an absence of infiltrations in any of the sections examined and prominent sinusoids in the livers of the PBS-injected hosts from both strains (Additional file 1: Figure S4).

**Figure 8:**
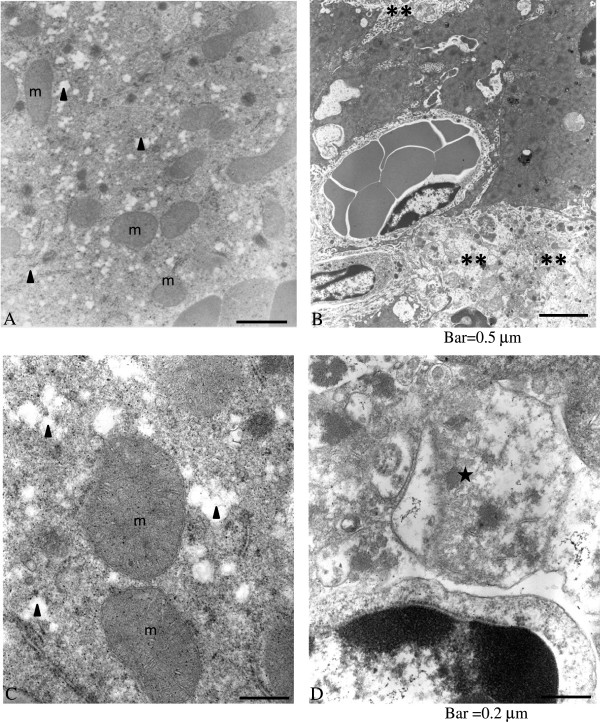
**Electron micrographs of sections of murine liver taken 12 hr after intravenous injection of 12 mg ConA/kg.** Representative samples shown are from C57Bl/6 **(B, D)** and C57Bl/6 *IFN-γ*^*−/−*^**(A, C)** mice. Note the areas of fibrosis (**) and deranged mitochondria (★) in the liver of C57Bl/6 mice. With the exception of fatty infiltrates (▲), the liver of the C57Bl/6 IFN-γ^−/−^ mice appears normal with intact mitochondria (m). Bars: A-B = 0.5 μm; C-D = 0.2 μm.

Electron micrographs of sections of murine liver taken 12 hr after intravascular injection of ConA (12 mg/kg) from C57Bl/6 and C57Bl/6 IFN-γ^−/−^ mice are shown in Figure 8. Areas of fibrosis and deranged mitochondria were prominent in the liver of C57Bl/6 mice. With the exception of fatty infiltrates, the liver of C57Bl/6 IFN-γ^−/−^ mice appeared normal - with intact mitochondria.

### FACS analyses of cellular isolates from liver

**Figure 9:**
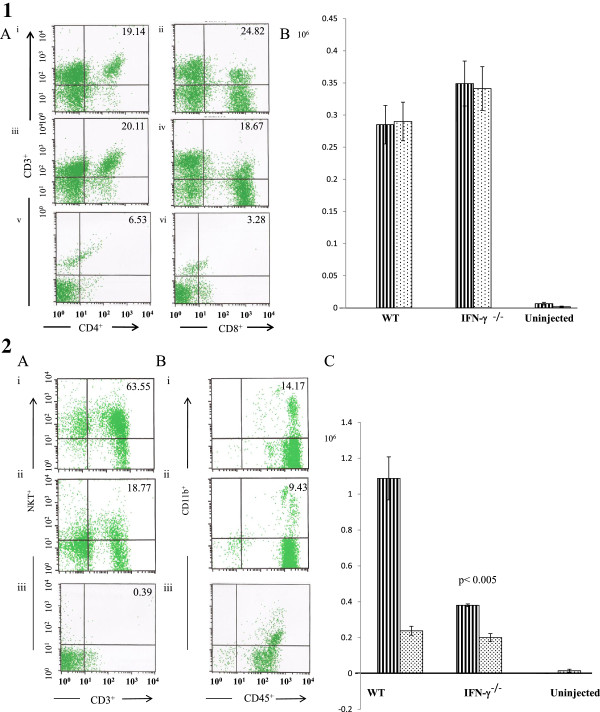
**FACS analysis.** Panel 1A (FACS analysis of CD3^+^CD4^+^ and CD3^+^CD8^+^ cells of liver samples): Representative liver samples from C57Bl/6 wild-type (i and ii) and IFN-γ^−/−^ on C57Bl/6 background (iii and iv) mice 12 hr after injection with 12 mg/kg of ConA. The number of mononuclear cells per mouse isolated from ConA injected mice was 1.5 × 10^6^ (C57Bl/6) and 1.8 × 10^6^ (C57Bl/6 IFN-γ^−/−^), respectively. The number of mononuclear cells per mouse isolated from uninjected C57Bl/6 mice was 5.0 × 10^4^ (v and vi). The values in the upper right corners (i-vi) are percents of CD4^+^ or CD8^+^ T cells. **Panel 1B:** The numbers (×10^6^) of CD4^+^ (lines) and CD8^+^ (dots) T cells for wild-type (WT) and IFN-γ^−/−^ mice are shown. There were no significant differences between the numbers of CD4^+^ or CD8^+^ T cells isolated from the two strains. As expected, there was near absence of these cells in the uninjected mice. **Panel 2A-B (FACS analysis of CD3**^**+**^**NKT**^**+**^**and CD45**^**+**^**CD11b**^**+**^**cells of liver samples)**. Note the significant number of NKT^+^ cells (lines) present in the WT mice compared to the IFN-γ^−/−^ mice (p<0.005). There was no significant difference between the numbers of CD11b^+^ cells (dots) isolated from the two strains. As expected, there was near absence of these cells in the uninjected mice.

FACS analyses of liver homogenates prepared from C57Bl/6 and C57Bl/6 IFN-γ^−/−^ mice were performed to characterize the phenotype of cellular infiltrates 12 hr after intravenous injection of ConA (12 mg/kg). As shown in Figure 9, T-cells (CD4^+^, CD8^+^) and macrophages (CD45^+^CD11b^+^) were present in both strains. NKT cells (CD3^+^NKT^+^), on the other hand, were significantly present only in the wild-type mice (p < 0.005). Of note, much lower number of cells was present in the uninjected C57Bl/6 mice.

## Discussion

Concanavalin A (ConA) is a potent inducer of inflammatory cytokines from the innate immune cells. Intravenous administration of this lectin results in progressive hepatic inflammation in mice [1,2]. The events commence within 2 hr of ConA treatment, with attachment of leukocytes to the hepatic endothelium and activation of NK cells and macro-phages. At about 8 hr, lymphocytes adhere to hepatocytes. At this stage, morphological changes (e.g., cell surface blebs) on hepatocytes and endothelium - and recruitment of neutrophils to the vascular lumen - become prominent. In later stages, the sinusoidal surfaces of hepatocytes show rupture of the cell membrane and loss of cytoplasm (necrosis) [1,2]. Thus, it appears that the ConA-induced hepatic injury represents deleterious effects of inflammatory molecules released by a number of cellular sources, including Kupffer cells, NK cells, leukocytes, and endothelial cells [12,13].

Changes in liver tissue bioenergetics during the course of ConA-induced hepatitis have not been previously studied. It is unknown whether the inflammatory cytokines alter hepatocyte metabolism (e.g., inducing higher energy expenditure and requirement). A novel finding in this study is that an increase in hepatocyte O_2_ consumption is observed 3 hr after ConA injection. This effect is not present in the C57Bl/6 IFN-γ^−/−^ mice, which has a milder disease. Cytokines, including IFN-γ, typically appear in the serum 2 to 3 hr after ConA administration [14]; the findings here are in keeping with those earlier studies (Figure 4).

A suppression of ConA-induced hepatitis is noted in the IFN-γ^−/−^ mice (Figures 6, 8 and Additional file 1: Figure S3). Nevertheless, the precise mechanism for the up-regulation of hepatocyte mitochondrial O_2_ consumption by ConA treatment is unknown. This effect was noted at 3 hr after ConA injection when the inflammatory cells were not yet prominent (Figure 6A).

Ac-DEVD-AMC is a synthetic substrate that enters the cells rapidly and is cleaved by caspases, yielding the fluorescent compound AMC [7]. Following cell disruption, any released AMC is separated via HPLC and detected by fluorescence with great sensitivity. The reduction in AMC peak areas due to the pan-caspase inhibitor zVAD-fmk confirms that intra-cellular caspases are responsible for the cleavage. As shown in Figure 5, the AMC peak was noted 6 hr after ConA injection and it was significantly reduced by zVAD-fmk; thus, ConA treatment led to caspase activation. Furthermore, the noted drop in hepatocyte O_2_ consumption 6 hr after ConA administration (see Figure 2) could be due to caspase-induced inhibition of the mitochondrial function. The more prominent decrease in hepatocyte respiration at 12 hr after ConA, on the other hand, was associated with areas of necrosis and deranged mitochondria (see Figures 6 and 8). Of note here, the hepatocyte nuclei and mitochondria remained relatively preserved 3 and 6 hr after ConA administration to the hosts (Figure 7). The pathologic changes in ConA-induced hepatitis are heterogeneous, likely accounting for the variability in the rate of O_2_ consumption observed here. Again, the precise mechanism for this patchy accumulation of inflammatory cells and focal necrosis is presently unknown.

IFN-γ is a key inflammatory cytokine in ConA-induced hepatitis [3]. The probable source of IFN-γ is NKT cells recruited into the liver, whose numbers were found to be significantly elevated in C57BL/6 mice compared with their knock-out counterparts 12 hr after ConA injection (Figure 9) [15-17]. In the studies here, the lack of IFN-γ in the C57BL/6 IFN-γ^−/−^ mice partially repressed the disease (Figure 8), even when a higher dose of ConA (20 mg/kg) was employed. Interestingly, liver tissue respiration was also less impacted in these mice. The progressive increases in serum IFN-γ, ALT, AST, and intracellular caspases in the ConA-treated C57Bl/6 mice emulated the progression of hepatocyte morphologic changes (confirmed by light and electron microscopy). Inexplicably, however, the ultrastructural changes at 3 and 6 hr after injection with ConA were unremarkable, and deranged mitochondria were noted only at 12 hr (Figure 8).

The observed enhancement of hepatocyte respiration in ConA hepatitis is not a direct action of IFN-γ (Additional file 1: Figure S3). It is due to the ConA-induced disease, in which IFN-γ contributes to its pathogenesis. For example, NKT cells (CD3^+^NKT^+^) were significantly present in the wild-type mice compared to IFN-γ^−/−^ mice (p < 0.005, Figure 9).

## Conclusion

In conclusion, the results of the current studies show increases in hepatocyte O_2_ consumption 3 hr after ConA treatment. This finding appears to reflect higher metabolic energy expenditure early in the course of disease.

## Competing interest

The authors claim no conflicts of interest. The authors alone are responsible for the contents of this manuscript.

## Authors’ contributions

MA, EMB and AKS designed the study, carried out the analysis, interpreted the data and drafted the manuscript. AS conceived of the study, measured respiration, ATP content and caspase activity. EMB examined histological and immunohistochemical staining. All authors read and approved the final manuscript.

## Pre-publication history

The pre-publication history for this paper can be accessed here:

http://www.biomedcentral.com/1471-230X/13/6/prepub

## Supplementary Material

Additional file 1 Figure S1 Typical runs of liver tissue respiration with cyanide and glucose oxidase are shown below. NaCN (10 mM) inhibited 82-86% of liver O_2_ consumption, confirming that the oxidation occurred in the mitochondrial respiratory chain. The remaining O_2_ in the solution was depleted with the addition of glucose oxidase (50 μg/ml). **Figure S2.** ConA did not significantly alter liver tissue respiration *in vitro*. A representative experiment is shown below. Briefly, murine liver specimens (4-13 mg) were incubated *in vitro* at 37^o^C with and without 10 μg/ml ConA in KH buffer (continuously gassed with 95% O_2_:5% CO_2_) for up to ~8 hr. Minute zero corresponds to time of sacrifice of the animal. Samples were alternatively removed from the incubation solution, rinsed with the same buffer, and placed in the instrument for O_2_ measurements at 37^o^C. Rates of respiration, *k* in μM O_2_ min^-1^ and *k*_*c*_ in μM O_2_ min^-1^ mg^-1^, are shown at the bottom of the runs. Similar results were observed with higher ConA concentrations (data not shown). **Figure S3.** IFN-γ inhibited liver tissue respiration *in vitro*. Liver specimens (~25 mg each) were incubated *in vitro* at 37^o^C in KH buffer continuously gassed with 95% O_2_:5% CO_2_. Samples were sequentially removed from the incubation solution and placed in the oxygen vial for measurement of respiration. Minute zero corresponds to time of sacrifice of the animal. At indicated time points, IFN-γ (100, 50 or 10 ng/mL), sodium cyanide (CN, 10 mM) or glucose oxidase (GO, 5 μg/mL) were added. Rates of respiration, *k* in μM O_2_ min^-1^, are shown. **Figure S4.** Micrographs of hematoxylin-stained paraffin embedded liver sections of uninjected (A) or PBS (C, at 12 hr) injected C57Bl/6 and uninjected (B) or PBS injected (D, at 12 hr) C57Bl/6 *IFNγ*^*−/−*^ mice. Note the absence of infiltrations in any of the sections and the prominent sinusoids in PBS injected livers of both strains. Bar = 50 μm. (DOC 4201 kb) Click here for file
