# Supplementary material for: Derangements of liver tissue bioenergetics in Concanavalin A-induced hepatitis
Source: BMC Gastroenterol. 2013 Jan 12;13:6. doi: 10.1186/1471-230X-13-6 (PMC3571906; doi:10.1186/1471-230X-13-6)
Supplement: Additional file 1 Figure S1 — Typical runs of liver tissue respiration with cyanide and glucose oxidase are shown below. NaCN (10 mM) inhibited 82-86% of liver O2 consumption, confirming that the oxidation occurred in the mitochondrial respiratory chain. The remaining O2 in the solution was depleted with the addition of glucose oxidase (50 μg/ml). Figure S2. ConA did not significantly alter liver tissue respiration in vitro. A representative experiment is shown below. Briefly, murine liver specimens (4-13 mg) were incubated in vitro at 37oC with and without 10 μg/ml ConA in KH buffer (continuously gassed with 95% O2:5% CO2) for up to ~8 hr. Minute zero corresponds to time of sacrifice of the animal. Samples were alternatively removed from the incubation solution, rinsed with the same buffer, and placed in the instrument for O2 measurements at 37oC. Rates of respiration, k in μM O2 min-1 and kc in μM O2 min-1 mg-1, are shown at the bottom of the runs. Similar results were observed with higher ConA concentrations (data not shown). Figure S3. IFN-γ inhibited liver tissue respiration in vitro. Liver specimens (~25 mg each) were incubated in vitro at 37oC in KH buffer continuously gassed with 95% O2:5% CO2. Samples were sequentially removed from the incubation solution and placed in the oxygen vial for measurement of respiration. Minute zero corresponds to time of sacrifice of the animal. At indicated time points, IFN-γ (100, 50 or 10 ng/mL), sodium cyanide (CN, 10 mM) or glucose oxidase (GO, 5 μg/mL) were added. Rates of respiration, k in μM O2 min-1, are shown. Figure S4. Micrographs of hematoxylin-stained paraffin embedded liver sections of uninjected (A) or PBS (C, at 12 hr) injected C57Bl/6 and uninjected (B) or PBS injected (D, at 12 hr) C57Bl/6 IFNγ−/− mice. Note the absence of infiltrations in any of the sections and the prominent sinusoids in PBS injected livers of both strains. Bar = 50 μm. (DOC 4201 kb) [file 1471-230X-13-6-S1.doc]

**SUPPLEMENT**

**Derangements of Liver Tissue Bioenergetics in Concanavalin A-Induced Hepatitis**

Mariam Al-Shamsi^1^, Allen Shahin^1^, Eric P. K. Mensah-Brown^2,*^, Abdul-Kader Souid^3,*^

Departments of Immunology^1^, Anatomy^2^, and Pediatrics^3^, College of Medicine and Health Sciences, United Arab Emirates University, Al Ain, Abu Dhabi, United Arab Emirates (UAE)

Correspondence: A.-K. S. and E. P. K. M.-B. (asouid@uaeu.ac.ae; tel. +9-713-713-7429; fax +9-713-767-2022; [ericb@uaeu.ac.ae](mailto:ericb@uaeu.ac.ae); tel. +9713-713-7495; fax +9713-767-2033)

**Running Title:** Liver Bioenergetics in ConA Hepatitis

**Keywords:** Concanavalin A (ConA), hepatitis, caspase, AST, ALT, IFN-γ

**Figure S1.** Typical runs of liver tissue respiration with cyanide and glucose oxidase are shown below. NaCN (10 mM) inhibited 82-86% of liver O_2_ consumption, confirming that the oxidation occurred in the mitochondrial respiratory chain. The remaining O_2_ in the solution was depleted with the addition of glucose oxidase (50 μg/ml).

|  |
| --- |

**Figure S2.** ConA did not significantly alter liver tissue respiration *in vitro*. A representative experiment is shown below. Briefly, murine liver specimens (4-13 mg) were incubated *in vitro* at 37^o^C with and without 10 μg/ml ConA in KH buffer (continuously gassed with 95% O_2_:5% CO_2_) for up to ~8 hr. Minute zero corresponds to time of sacrifice of the animal. Samples were alternatively removed from the incubation solution, rinsed with the same buffer, and placed in the instrument for O_2_ measurements at 37^o^C. Rates of respiration, *k* in μM O_2_ min^-1^ and *k_c_* in μM O_2_ min^-1^ mg^-1^, are shown at the bottom of the runs. Similar results were observed with higher ConA concentrations (data not shown).

**Figure S3.** IFN-γ inhibited liver tissue respiration *in vitro*. Liver specimens (~25 mg each) were incubated *in vitro* at 37^o^C in KH buffer continuously gassed with 95% O_2_:5% CO_2_. Samples were sequentially removed from the incubation solution and placed in the oxygen vial for measurement of respiration. Minute zero corresponds to time of sacrifice of the animal. At indicated time points, IFN-γ (100, 50 or 10 ng/mL), sodium cyanide (CN, 10 mM) or glucose oxidase (GO, 5 μg/mL) were added. Rates of respiration, *k* in μM O_2_ min^-1^, are shown.

**Figure S4.** Micrographs of hematoxylin-stained paraffin embedded liver sections of uninjected (A) or PBS (C, at 12 hr) injected C57Bl/6 and uninjected (B) or PBS injected (D, at 12 hr) C57Bl/6 *IFNγ^−/−^* mice. Note the absence of infiltrations in any of the sections and the prominent sinusoids in PBS injected livers of both strains. Bar = 50 μm
